# Unraveling dispersion and buoyancy dynamics around radial *A* + *B* → *C* reaction fronts: microgravity experiments and numerical simulations

**DOI:** 10.1038/s41526-024-00390-8

**Published:** 2024-05-09

**Authors:** Yorgos Stergiou, Darío M. Escala, Paszkál Papp, Dezső Horváth, Marcus J. B. Hauser, Fabian Brau, Anne De Wit, Ágota Tóth, Kerstin Eckert, Karin Schwarzenberger

**Affiliations:** 1 Institute of Fluid Dynamics, Helmholtz-Zentrum Dresden-Rossendorf, Bautzner Landstr. 400, 01328 Dresden, Germany; 2 Institute of Process Engineering and Environmental Technology, Technische Universität Dresden, 01062 Dresden, Germany; 3 Nonlinear Physical Chemistry Unit, Service de Chimie Physique et Biologie Théorique, Faculté des Sciences, Université Libre de Bruxelles (ULB), CP 231, 1050 Brussels, Belgium; 4 Department of Physical Chemistry and Materials Science, University of Szeged, Rerrich Béla tér 1., Szeged, Hungary; 5 Department of Applied and Environmental Chemistry, University of Szeged, Rerrich Béla tér 1., Szeged, Hungary; 6 Faculty of Natural Science, Otto-von-Guericke-Universität Magdeburg, Universitätsplatz 2, 39106 Magdeburg, Germany

**Keywords:** Fluid dynamics, Chemical physics, Chemical engineering

## Abstract

Radial Reaction–Diffusion–Advection (RDA) fronts for *A* + *B* → *C* reactions find wide applications in many natural and technological processes. In liquid solutions, their dynamics can be perturbed by buoyancy-driven convection due to concentration gradients across the front. In this context, we conducted microgravity experiments aboard a sounding rocket, in order to disentangle dispersion and buoyancy effects in such fronts. We studied experimentally the dynamics due to the radial injection of A in B at a constant flow rate, in absence of gravity. We compared the obtained results with numerical simulations using either radial one– (1D) or two–dimensional (2D) models. We showed that gravitational acceleration significantly distorts the RDA dynamics on ground, even if the vertical dimension of the reactor and density gradients are small. We further quantified the importance of such buoyant phenomena. Finally, we showed that 1D numerical models with radial symmetry fail to predict the dynamics of RDA fronts in thicker geometries, while 2D radial models are necessary to accurately describe RDA dynamics where Taylor–Aris dispersion is significant.

## Introduction

Reaction–Diffusion (RD) fronts have long been of interest to the scientific community due to the wide field of phenomena they describe. A subcategory of RD fronts, those due to the interplay of bimolecular *A* + *B* → *C* reactions and diffusion, are used to model numerous problems in chemical technology^1,2^; finance^3^; linguistics^4^; particle physics^5^, among others. Such fronts have been studied theoretically^6^ and their corresponding scalings were confirmed experimentally^7–9^.

When combined with radial advection under flow conditions, the complexity of the resulting Reaction–Diffusion–Advection (RDA) systems is significantly increased. Although used to model current technological applications in combustion^10^; engineering geology^11^; selective production of precipitates^12^ and carbon capturing technologies^1^, RDA front dynamics are yet to be fully described.

**Fig. 1:**
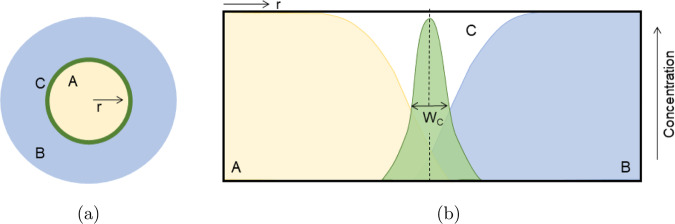
Sketch of a Reaction–Diffusion–Advection *A* + *B* → *C* front with radial symmetry. **a** Schematic representation of the front in top view. **b** The concentration distribution of reactants *A* and *B* and product *C* and the qualitative description of the front width, *W*_*C*_.

Recently, modeling has focused on deriving scalings of RDA fronts where a solution of *A* is radially injected at a constant flow rate in a pool of solution *B*. Such a radial RDA front is depicted in Fig. 1. A top view of the radial geometry is provided in Fig. 1a, whereas the radial reactant and product distribution is sketched in Fig. 1b. The sketch further includes the front width, *W*_*C*_, which is an important observable in radial RDA fronts. The RDA dynamics has been modeled using either a 1D theory assuming a plug flow and polar symmetry^13–15^, 2D radial theories accounting for the Poiseuille flow profile^16^ and Taylor–Aris dispersion^17,18^ with radial symmetry^19^ or in 3D with spherical symmetry^20,21^.

Nevertheless, experimental validation of models in radial RDA fronts is still lagging behind, as past experiments that were carried out in Hele-Shaw (HS) cells^13,22^ are bound to buoyant effects that distort the overall advection dynamics because of the slight density differences between the solutions of reactant *A* and *B* and product *C* ^23^. Even if the HS cell as a quasi two dimensional, horizontally arranged configuration is able to strongly reduce buoyant effects, Taylor–Aris dispersion effects could not be isolated in these systems. This is due to the fact that a certain range of fluid gap heights and flow rates must be covered to capture different scenarios of front dynamics. Microgravity experiments already were successfully employed in the literature to separate diffusion and advection from gravity-driven convection^24,25^, or for more complex combustion reactions including thermal effects^26–28^.

Hence, to address the mentioned deficiencies, we conducted *A* + *B* → *C* RDA experiments in a radial geometry under absence of gravitational acceleration, aboard a Sounding Rocket (SR), attempting to disentangle the influence of buoyancy from the general RDA dynamics and to obtain experimental data that are otherwise not accessible on Earth. In parallel, we performed numerical simulations, based on the model by ref. ^16^ to showcase the necessity of considering 2D effects (i.e., Taylor–Aris dispersion) when modeling radial RDA fronts, which cannot be described adequately with the use of simplified 1D models^13–15^.

Both experimental and numerical results are shown, compared and discussed in section Results and Discussion, together with concluding remarks in section Conclusions. Details on the experiments conducted under microgravity conditions onboard a sounding rocket are presented in section Sounding Rocket Experiment. The procedure of the numerical simulations is included in section Numerical Simulations.

## Results and discussion

### Overview

The sounding rocket experiments comprise three reactors with different gap height values and injection flowrates. They are presented together with their corresponding ground reference runs. More details on the experimental parameters and procedures are included in section Methods.

**Fig. 2:**
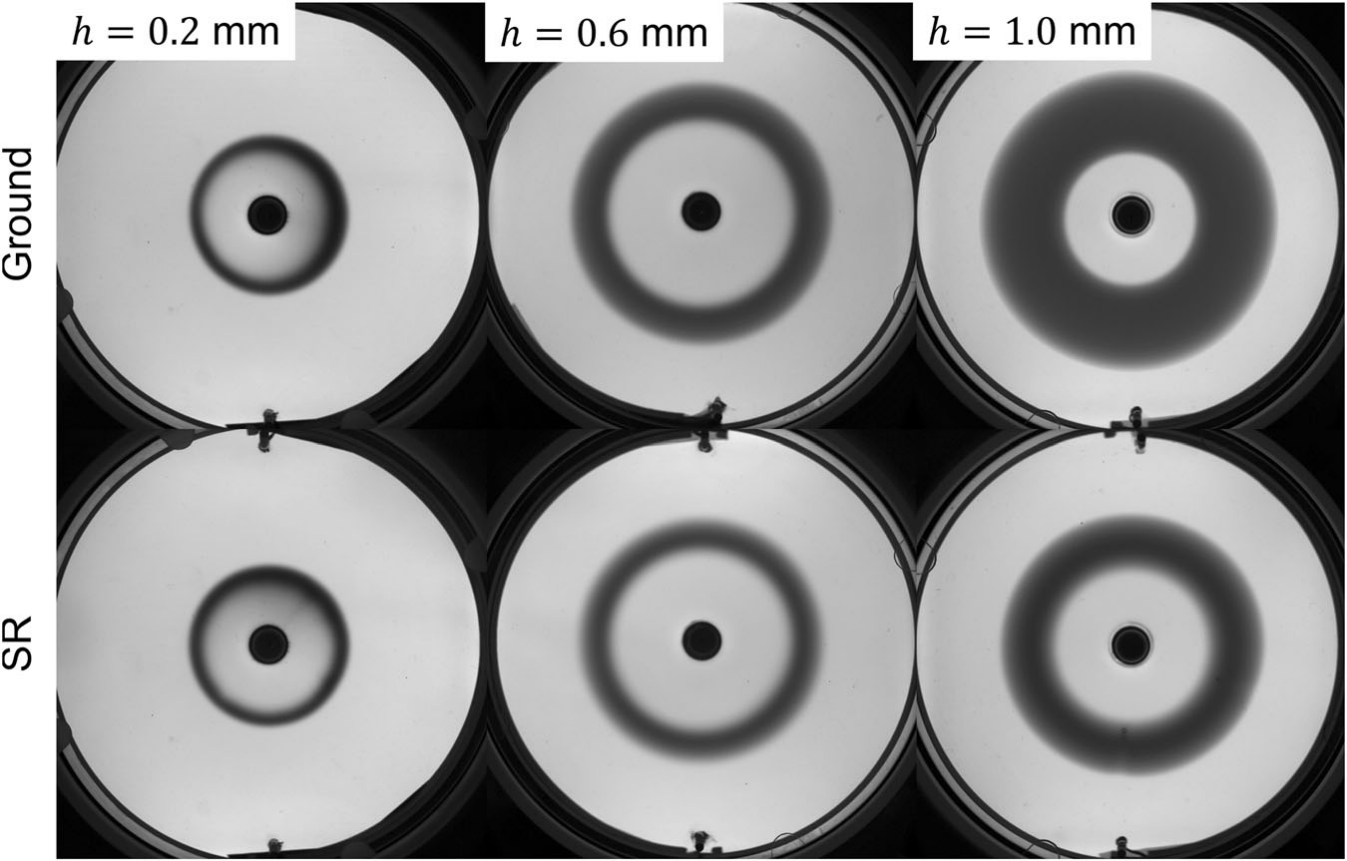
Spatial distribution of the product *C.* Experiments on ground (upper row) and during microgravity (lower row) at *t* = 150 s, for all three gap height experiments (*h* = 0.2, 0.6 and 1.0 mm).

In Fig. 2, the comparison between ground and SR cases for all three gap heights *h* and flow rates *Q* (given in Table 1) is shown at time *t* = 150 s after the start of the injection. The differences between ground and SR cases are clearly visible for the experiments with *h* = 0.6 and 1.0 mm, i.e., the front width *W*_*C*_ is significantly larger on ground in these cases. The difference in *W*_*C*_ strongly grows as *h* increases. In line with this, no difference is observed visually for the smaller gap height (*h* = 0.2 mm). More experimental images are provided in the Supplementary Information.

**Table 1:** Experimental conditions for the different sounding rocket experiments with varied gap height, *h*, and flow rate, *Q*

Experiment	*h* (mm)	*Q* (mL min^−1^)
1	0.2	0.05362
2	0.6	0.3217
3	1.0	0.5362

### Total amount of product *C*, ${\overline{n}}_{C}$

**Fig. 3:**
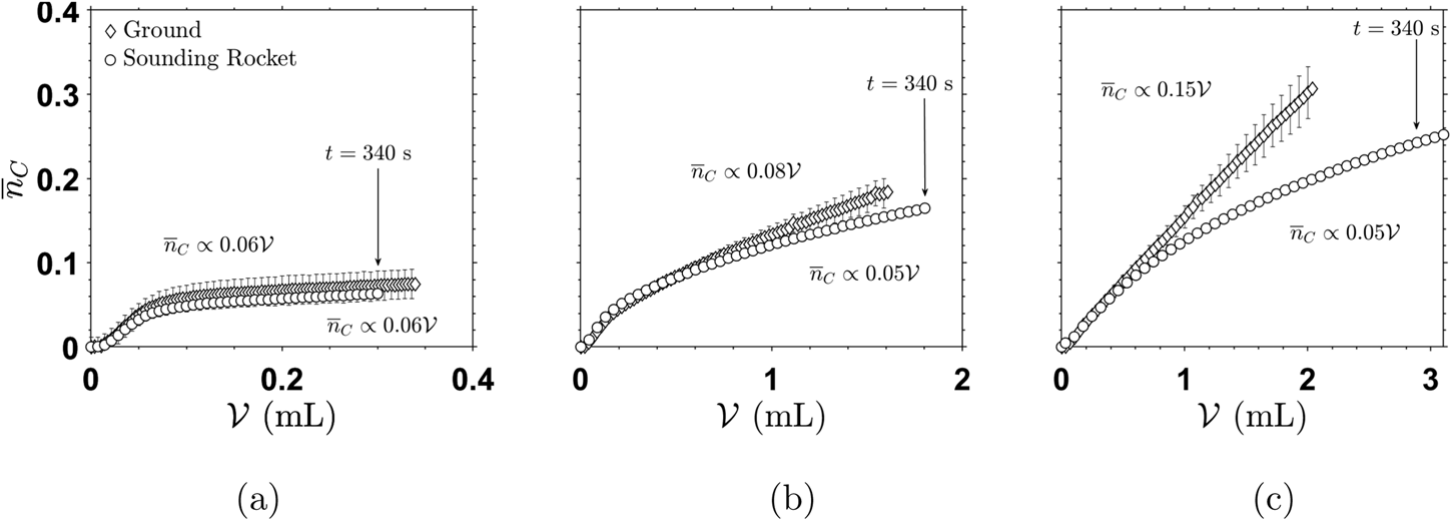
Normalized amount of product, ${\overline{n}}_{C}$, as a function of volume injected, ${{{\mathcal{V}}}}$. **a** Gap height *h* = 0.2 mm, **b** 0.6 mm and **c** 1.0 mm. The graphs for the three different gap heights juxtapose the sounding rocket and ground experiments. The time *t* = 340 s is marked as a reference time for all experiments, since different volumes of A are injected for different *h*. The error bars for the ground values represent the standard deviation among all ground experiment repetitions. The *y*−axis values are the same for all subfigures.

In Fig. 3, the comparison between the normalized total amount of product, ${\overline{n}}_{C}$, generated on ground and during the SR experiment is plotted against the total volume of reactant injected, ${{{\mathcal{V}}}}$, for all three gap heights.

As the volumetric flow rate, *Q*, remained constant throughout the full duration of each experiment, the volume injected grows linearly with time: ${{{\mathcal{V}}}}=Qt$.

It is directly visible that ${\overline{n}}_{C}$ reaches higher values in all experiments when conducted on ground. However, for *h* = 0.2 mm (Fig. 3a), this difference is within the experimental variation as, for the thinnest gap, it is expected that the buoyant effects will not dominate on ground. With increasing *h* (Fig. 3b, c) the difference is much more pronounced, i.e., buoyancy forces lead to a significantly increased product generation for the ground cases.

Since the time duration for all gap heights is comparable (time stamp at *t* = 340 s marked in Fig. 3), we can conclude that more product is generated for larger *h* in the same time, both on ground and in micro-g. In terms of dimensional amount of product, this seems logical, since a higher amount of product can be formed in a larger reactor volume of finite radius, *r*_*max*_ (cf. Equation (3)), and a larger amount of reactant *A* is injected which can react to produce *C*. Nevertheless, this even holds for the normalized amount of product shown in Fig. 3, which relates the measured amount of product to the case when the reactor is completely filled with a solution of maximum product concentration (i.e., a 1:1 fully mixed solution of *A* and *B*).

To better quantify the additional effect of buoyancy, the final slope of the curves (product generation rate) was obtained by fitting with a linear relation, ${\overline{n}}_{C} \sim \alpha {{{\mathcal{V}}}}$, where *α* is a constant. The resulting coefficients are plotted in Fig. 3 and summarized in Table 2.

**Table 2:** Variation of coefficient *α* for generation of product ${\overline{n}}_{C}$

Experiment	*h* (mm)	*α*_*SR*_ (mL^−1^)	*α*_*Ground*_ (mL^−1^)
1	0.2	0.06	0.06
2	0.6	0.05	0.08
3	1.0	0.05	0.15

The obtained production rates confirm that the influence of buoyancy seems to be negligible for the experiment with *h* = 0.2 mm. For the experiments with *h* = 0.6 and 1.0 mm, *α*_Ground_ increases with increasing *h*, from 0.08 to 0.15 mL^−1^. For the SR experiments, a value of *α*_*SR*_ = 0.05 mL^−1^ is found for both experiments with larger *h*, which is also close to *α* = 0.06 mL^−1^ in the 0.2 mm experiment.

**Fig. 4:**
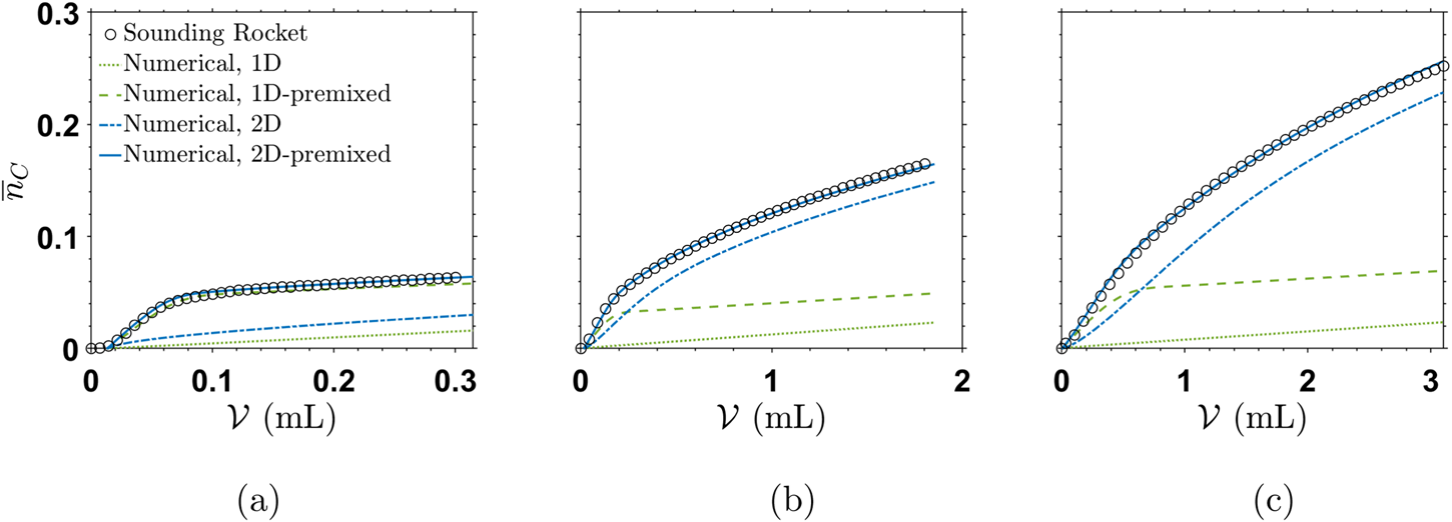
Normalized amount of product, ${\overline{n}}_{C}$, progression with volume injected, ${{{\mathcal{V}}}}$, in experiments and simulations. **a** Gap height *h* = 0.2 mm, **b** 0.6 mm and **c** 1.0 mm. The *y*−axis values are the same for all subfigures.

Owing to the absence of buoyancy effects, the micro-g experiments can now be compared to the 2D dispersion model^16^ and the simplified 1D approach^13,14^. For both 1D and 2D cases, additional simulations are performed that consider the premixed concentration of product which already forms in the inlet valve of the HS cells, cf. section Numerical Simulations. In Fig. 4, the ${\overline{n}}_{C}$ curves obtained by the numerical simulations are presented against the corresponding SR microgravity experimental results. For all three cases, a good agreement is found between the experimental data and the numerical values obtained by the 2D simulations that additionally account for the premixing effect.

For the experiment with *h* = 0.2 mm, both the 1- and 2D approaches including premixing adequately predict the progression of ${\overline{n}}_{C}$. A slight underestimation is observed for the 1D premixing case which is a direct effect of the absence of hydrodynamic mixing because of the missing Taylor–Aris dispersion. Nevertheless, the 1D curve with premixing can still serve as an adequate approximation of the product formation in the smallest gap. This also points towards the fact that the Poiseuille profile is rather negligible for small *h*. Consequently, hydrodynamic models that ignore wall friction can indeed be used for cases with low *h*, that is in cases where diffusive mass transfer dominates and equilibrates the concentration gradients on the *z*-direction fast enough. This applies for high values of diffusion coefficients (*D*) or small geometrical lengthscales over which diffusion needs to act (in this case, *h*). Both parameters are contained in the local Péclet number, *Pe*_*L*_, in Equation (2), see section Sounding Rocket Experiment. However, the models without premixing effects strongly deviate from the experimental results for the 0.2 mm cell (Fig. 4a). This is expected, as a certain volume of product solution has a bigger effect on ${\overline{n}}_{C}$ in cells with smaller *h*, i.e., in cells with smaller volume. Since dynamics in the HS cell with the smallest gap size is very sensitive to premixing at the experimental inlet conditions, additional CFD simulations are performed considering the actual flow field in the inlet plug geometry (see Supplementary Information). These simulations confirm that slight setup imperfections at the inlet might lead to this notable effect in relation to the small liquid volume within the thinnest HS gap.

**Fig. 5:**
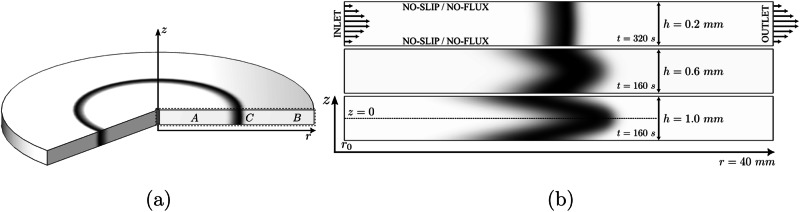
Numerical domain and simulation parameters. **a** The 2-dimensional axisymmetric domain represents a simplification of a 3-dimensional system with radial symmetry. **b** Product distribution for *h* = 0.2, 0.6 and 1.0 mm, with corresponding flow rate given in Table 1, at times *t* = 320, 160 and 160 s, from top to bottom. The aspect ratio of the cells is altered for the sake of visual clarity and ease of comparison between the front shapes.

For the two bigger gap height reactors (*h* = 0.6 and 1.0 mm), the 1D model (either including premixing or not) severely underestimates ${\overline{n}}_{C}$, validating the assumption that for increasing *h*, a 2D model is needed to describe the dispersion-dominated dynamics. This is further supported by classical Taylor–Aris dispersion derivations^22,29^, where an increase in Péclet number leads to increased prevalence of Taylor–Aris dispersion (i.e., an increase in the effective diffusion coefficient). This can also be visualized in Fig. 5b, as the front with *h* = 0.2 mm shows a much more uniform concentration profile on the *z*-direction.

The plain 2D model without premixing shows qualitative agreement in the product curve progression, and reproduces a good quantitative approximation for the amount of product generated in the two experiments with bigger *h* (Fig. 4b, c). Nevertheless, ${\overline{n}}_{C}$ is again slightly underestimated, because no initial premixed volume, containing a certain amount of *C* is included. This effect is significantly less pronounced compared to *h* = 0.2 mm, as the premixed amount of product remains of the same order whereas the total reactor volume gets significantly bigger with increasing *h*. Thus, the concentration in *C* that initially enters the reactor is small compared to the total amount of product *C* generated in experiments with large *h*.

### Front width, *W*_*C*_

**Fig. 6:**
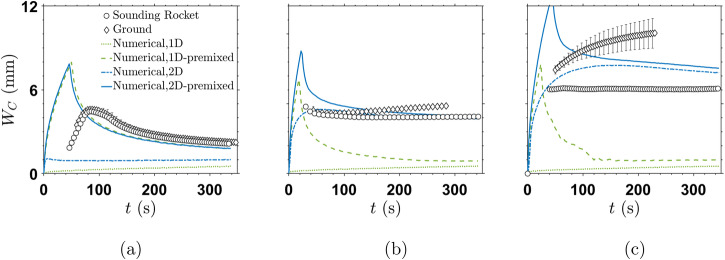
Front width, *W*_*C*_, temporal evolution for sounding rocket, ground experiments and numerical simulations. **a** Gap height *h* = 0.2 mm, **b** 0.6 mm and **c** 1.0 mm. The y−axis values are the same for all subfigures. The error bars for the ground values represent the standard deviation among all ground experiment repetitions.

Figure 6 presents the evolution of the front width, *W*_*C*_, for all sounding rocket and ground experiments together with the complete set of simulation results. In line with the results in the previous section, the experiments are strongly affected by gravity at larger gap heights. The dynamics on ground or in microgravity are clearly different for *h* = 1 mm (as already visible in Fig. 2), while the related curves overlap within the range of experimental uncertainty for *h* = 0.2 mm.

At the two larger *h* ground experiments, *W*_*C*_ continuously increases in time as a direct effect of the additional front growth caused by the gravity current due to the density difference between the reactant solutions. As a result of the wider fronts for larger *h*, the ground experiments can only be evaluated for a much shorter time, since the front tip reaches the rim of the reactor earlier because of the additional convection. This gravity influence severely obscures the original front dynamics, which is further discussed in the following.

A feature of all microgravity experiments and numerical simulations - except the 1D model without premixing - is the larger front width in the initial phase which subsequently decreases and reaches an almost constant value. This feature is most pronounced in the simulation models including premixing, which helps to understand the general mechanism of the front width decrease. If an existing radial reaction front (containing a certain amount of product) moves with the injection flow, then due to its increasing circumference it becomes thinner, as it moves towards the outer regions of the HS cell. The product can either be present due to the premixing in the inlet plug, or due to the previous product formation in the radial reaction front evolving within the HS cell. If this thinning effect dominates over the front growth caused by the species mass transfer and ongoing reaction, the front width decreases, as previously observed in numerical studies^16^. The same mechanism applies for the 2D simulations without premixing, as the continuing injection also leads to a stretching of the previously formed product front. The decrease is stronger for the cases where premixing has more influence, i.e., for smaller gaps. Moreover, the simple premixing model in the simulations assumes that the premixed fluid initially enters the HS fluid gap already with the maximum premixed concentration (*C*_*p*,*max*_, cf. section Numerical Simulations). In the experiments, the premixed product concentration might be increasing gradually until the main body of premixed volume has entered the HS cell, leading to the reduced initial spikes in *W*_*C*_. For larger *h*, the premixing effect is strongly reduced as discussed before. Hence, a significant part of the initial spike is hidden in the time span until the wide front is completely detached from the region which is optically covered by the central inlet valve.

At later times, the front width converges towards a constant value, which is *W*_*C*_ ≈ 2 mm, 4 mm and 5.5 mm for the microgravity experiments with 0.2 mm, 0.6 mm and 1.0 mm gap height, respectively. As the distance between the front and the central inlet increases, the local velocities are significantly reduced due to the radially diverging flow. Hence, diffusive mixing across the front gains in importance which compensates for the thinning effect. At long enough times, beyond the front thinning and plateau region, diffusion would prevail and *W*_*C*_ would start growing again. However, this region is beyond the duration of the microgravity experiments.

Similar to the amount of generated product, the best agreement is found for the 2D model including the premixing effect in the inlet plug. For the smallest gap, the 1D model with premixing is likewise applicable. For larger *h*, also the plain 2D model yields reasonable agreement due to the reduced premixing influence in the experiments. Comparing the 2D simulations without premixing at different *h*, the curves approach a constant value already at earlier times for smaller *h*, since diffusive mass transfer in the *z*-direction blurs any concentration gradients faster. In such cases, diffusion compensates for the thinning effect faster and subsequently the front will enter a steady diffusion–dominated regime sooner. This aspect of Taylor–Aris dispersion progress is also theoretically derived in classical formulations^29^. The largest gap *h* = 1.0 mm shows the most significant deviation to the numerical results at later times. This can be explained by the fact that the calibration curve (cf. Supplementary Information) is already close to saturation at higher concentrations for this thicker liquid layer, which leads to higher uncertainties in the *W*_*C*_ evaluation.

Although the 1D model predicts a continuous growth of *W*_*C*_ with time (as observed in the ground cases for *h* = 0.6 and 1.0 mm), it completely neglects the above-discussed dominant mechanisms of front stretching and the transition from Taylor–Aris dispersion dominated regimes towards more diffusion-dominated regimes, making it inadequate to model such cases. Furthermore, the 1D model severely underestimates the magnitude of the *W*_*C*_ values and predicts similar *W*_*C*_ values irrespective of *h*. Lastly, in the microgravity experiments, the decrease in *W*_*C*_ for reactors with bigger *h* could be studied, an observation otherwise not accessible on ground. Hence, only the microgravity experiments can clearly show the need to include Taylor–Aris dispersion, and can rule out the usage of 1D models for such systems, especially in cases with high *h*.

### Conclusions

We have investigated Reaction–Diffusion–Advection fronts in radial Hele-Shaw reactors under microgravity conditions onboard a sounding rocket. We conducted reference experiments on ground with identical reactors (in horizontal orientation) and identical parameter values. In addition, we performed corresponding numerical simulations using 1D and 2D models for the radial RDA fronts. Microgravity allowed us to isolate the effect of dispersion on the dynamics of the fronts, which is hardly feasible on ground. The experiments with larger gap heights show a significantly reduced amount of product generation and smaller reaction front width values under microgravity compared to the ground case. The numerical simulations were able to reproduce the evolution of the front in absence of buoyancy. This was achieved by including the dispersion effects arising from the parabolic velocity profile in the gap of the cell and a simple approach to consider premixing effects. Our findings on the effect of the premixing at the reactor inlet in relation to the reactor volume can be used as a guide for future studies in HS reactors, as this geometry is widely used for fluid dynamic model experiments^30^.

Indeed, our findings confirm that, in very small gaps with slow flow, 1D models can be employed. This is attributed to the fact that smaller length scales lead to a diffusion dominated regime much sooner, and the concentration profile across the gap height becomes uniform. However, such situations where both dispersion and buoyancy effects are negligible have only limited applicability. As can be seen from our results, the front dynamics under such conditions are very different from those in systems dominated by Taylor–Aris dispersion, and therefore the 1D models fail to reproduce the RDA fronts at larger gap heights.

The 2D models including dispersion could be validated by our microgravity experiments. Hence, the corresponding scalings^16^ can be applied to predict the product formation for various applications, since such reaction fronts are widely occurring in Nature and technology. Systems where Taylor–Aris dispersion is important but buoyancy effects can be neglected are for example very dilute systems, systems with small *h* and low solute diffusivity, highly viscous fluids, or space applications where gravity is absent^25^. Our findings provide a basis for further development of dispersion models in the direction of combustion or porous media flows^31^.

## Methods

Microgravity experiments in radial RDA fronts were carried out in the frame of the European Space Agency project “Chemically-Driven Interfacial Convection 4" (CDIC-4), using the TEXUS sounding rocket (SR) platform. Corresponding simulations aimed at testing the range of validity of the models and showcasing the need of a 2D approach for the study of similar RDA cases.

### Sounding rocket experiment

The CDIC-4 experiment was conducted onboard the TEXUS-57 sounding rocket mission, launched from the Esrange Space Center in Kiruna, Sweden. The experimental setup used in the sounding rocket module was designed and constructed by AIRBUS Defense and Space (Bremen, Germany). The design concept and experimental procedure were based on experiments previously conducted onboard a parabolic flight^32^ and on ground^22^.

As a model system for the *A* + *B* → *C* reaction, the formation of the complex FeSCN^2+^ (product C) from potassium thiocyanate KSCN (reactant A) and iron(III) nitrate Fe(NO_3_)_3_ (reactant B) was used. The experiments were performed in quasi two-dimensional planar geometries using a Hele-Shaw (HS) cell design with central injection.

The solutions were prepared from reagent-grade chemicals (Sigma-Aldrich). The HS cells were filled with a 0.03 mol L^−1^ Fe(NO_3_)_3_ aqueous solution (solution B, *ρ*_*B*_ = 1007.4 kg m^−3^ and *μ*_*B*_ = 1.08 mPa s), adjusted to acidic conditions (pH = 1) with HNO_3_. Subsequently, a 0.03 mol L^−1^ KSCN solution (solution A, *ρ*_*A*_ = 996.6 kg m^−3^ and *μ*_*A*_ = 0.97 mPa s) was injected into the HS cell through the central inlet at a constant flow rate, *Q*. When the two solutions come in contact, the brown-colored monocomplex FeSCN^2+^ is produced dominantly as product *C* ^33^ in the used solution compositions, according to the reaction scheme:

1 $${{{{\rm{Fe}}}}}^{3+}+{{{{\rm{SCN}}}}}^{-}\longrightarrow {{{{\rm{FeSCN}}}}}^{2+}.$$

By mixing equal volumes of solutions *A* and *B* the product solution *C* is obtained at maximum concentration, as the reaction is quasi-instantaneous. The density of this product solution is measured to be *ρ*_*C*_ = 1002.8 kg m^−3^ and the viscosity *μ*_*C*_ = 0.97 mPa s. The small viscosity differences between the solutions used, combined with the low flow rates, prevent the emergence of viscous fingering during the experiment^32^. The physical properties (density, viscosity) of the two reactant solutions (*A*, *B*) and of the resulting fully mixed product solution (*C*) were measured with an Anton Paar SVM 3001 pycnometer-viscosimeter (Anton Paar GmbH, Graz, Austria) at 20 ^*°*^C.

**Fig. 7:**
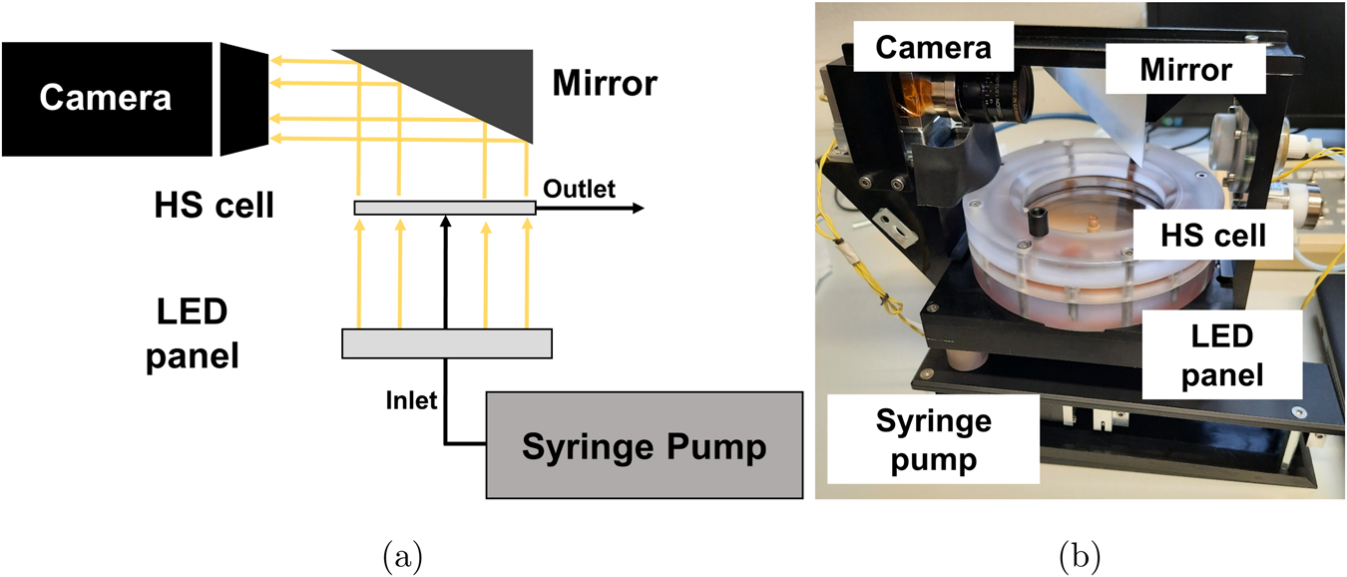
Experimental setup. **a** Sketch of setup principle. **b** Photo of one of the three experimental units used onboard the TEXUS 57 sounding rocket and for the ground reference tests.

**Fig. 8:**
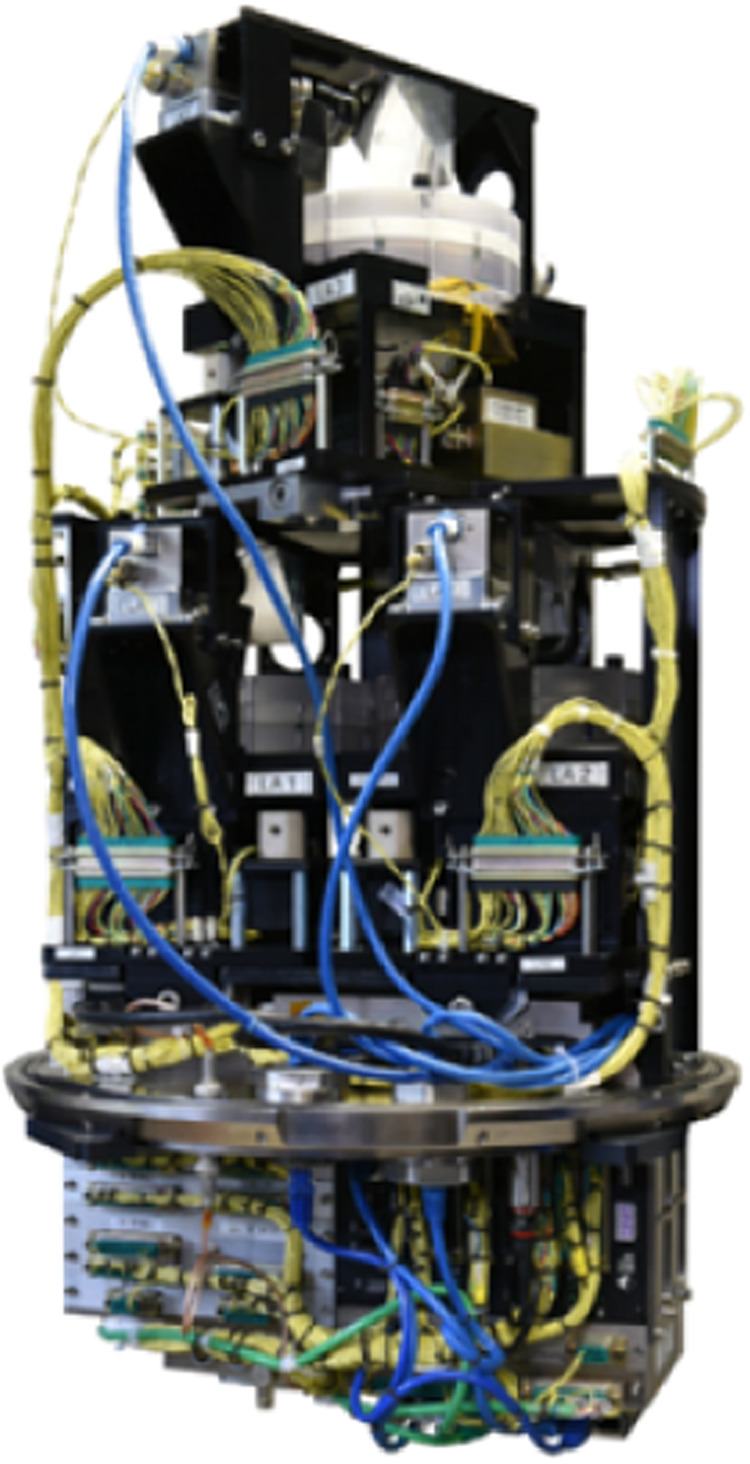
Photograph of the CDIC-4 module. The experimental units are visible, one on top and two at the bottom. The electronics compartment is also visible at the very bottom part of the image. The image is a courtesy of AIRBUS Defense and Space.

The main part of the experimental setup (Fig. 7) is the circular HS cell. The cell consisted of two parallel circular plates, a bottom plate made from quartz glass and a top plate of sapphire glass. The overall observable radius (*r*_*max*_) was 40 mm. The distance between the two plates could be set to different gap height values (*h*) using spacers laser-cut from a polytetrafluoroethylene (PTFE) foil. A central inlet tube was attached to the bottom plate of the HS cell via a solenoid valve mechanism (SVT-2, Tagasako Fluidic Systems, Nagoya, Japan). For the injection of the reactive solution, a syringe pump (SMC01 Precision-SY, SPETEC GmbH, Erding, Germany) was integrated into the metal frame structure of the experimental unit. Gastight glass Hamilton 1001, 1005 or 1010 syringes (Hamilton Company, Reno, NV, USA) were used in combination with the syringe pump. The liquid handling system consisted of PTFE tubing (Bohlender GmbH, Gruensfeld, Germany) with nominal inner diameter of 0.8 mm. Two outlet tubes were placed on the outer rim of the HS cell leading the exhaust liquid to a waste container (Fig. 8).

The reaction front was visualized by homogeneous illumination from below via a white light LED array (LTPVR100-00-1-W-24V-AEQ01, Opto Engineering, Mantova, Italy). The light emitted from the LED panel passes through the HS cell where it is partially absorbed by the reaction product. A mirror mounted on the metal structure and a GigE monochrome CMOS camera, 4096px × 3000px (cA4112-8gm, Basler AG Ahrensburg, Germany) together with an objective lens (CNG 16/1.8, Schneider Kreuznach, Bad Kreuznach, Germany) were used to take images throughout the experiment, with a rate of ≈ 6 Hz. A blue filter (Schneider BP 465-70 HT, Jos. Schneider Optische Werke GmbH, Bad Kreuznach, Germany) was inserted for the small gap height HS cell (i.e., *h* = 0.2 mm), to enhance the signal received by the camera. In addition, two temperature probes were integrated close to the rim of each of the HS cells in order to detect spatiotemporal temperature gradients that might affect the front dynamics. The temperature difference between both probes always was smaller than 0.2 K, and the temperature difference throughout the experimental run did not exceed 3.5 K. Ground reference experiments were performed with the same hardware used on the sounding rocket.

The experimental parameters for the three experiments conducted under microgravity are shown in Table 1. In addition to the one-time SR experiments, ground reference experiments have been conducted at least 3 times in repetition runs, with exactly the same values of parameters. The average values of all the observables are shown in the results section, along with their respective standard deviation. To better quantify the time- and position-dependent contribution of diffusion/convection in the different experiments, a local Péclet number, *Pe*_*L*_, is used:

2 $$P{e}_{L}=\frac{h{v}_{L}}{{D}_{C}},$$

where *D*_*C*_ is the diffusion coefficient of the product, *C*, equal to 4 × 10^−10^ m^2^ s^−1^ ^22^, and *v*_*L*_(*r*) = *Q*/(2*πhr*) the local average velocity. For experiment 2 and 3, the ratio *Q*/*h* is equal. This means that at the same radial distance, *r*, (which is reached at the same time, *t*), both experiments have the same *v*_*L*_, and *Pe*_*L*_ is proportional to *h*. Also global forms of the *Pe* number were used in previous studies to derive scaling relations, e.g., in^34^. It is defined as *Pe* = *Q*/(2*πhD*_*C*_) which is independent of *r*.

The TEXUS-57 sounding rocket was launched from the Esrange Space Center in Sweden on October 1st, 2022, at 08:26 (UTC+1). After the launch, the rocket followed a sub-orbital parabolic trajectory. Following the exit from the lower atmosphere, the motor detached, the payload was stabilized (de-spinned) and the free fall phase was initiated, providing high-quality microgravity (*O*(10^−5^) g) for the experimental payload. Microgravity was achieved 65 s after launch and the high-quality zero-g signal was received at 72 s after launch. The apogee of the flight at 239.02 km height was reached after 250.9 s. The end of the high-quality micro-g phase was at 421 s after launch, providing 349 s of experimental microgravity time. The CDIC-4 experiment was initiated 65 s after launch with the opening of the inlet valves. The experimental sequence was terminated successfully 450 s after launch. After that, the payload passed the re-entry phase and landed on hard ground at approximately 100 km from the launch site. The payload and the experimental cells were retrieved in good condition ca. 2 h after the lift-off.

#### Image processing

From the acquired experimental images, the spatial and temporal concentration distribution of the product, *C*(*x*, *y*, *t*), could be obtained using a similar procedure as described in ref. ^32^. In this procedure, the concentration of product *C* is calculated using the local pixel intensity values in the image sequence recorded by the camera. The calibration curves for the conversion of pixel intensity to concentration are provided in the Supplementary Information. It is then possible to obtain information about the total amount of product, *n*_*C*_(*t*), the normalized total amount of product, ${\overline{n}}_{C}(t)$, the radial concentration profile, *C*(*r*, *t*), and the reaction product width, *W*_*C*_(*t*), which is defined as the full-width at half-maximum (*FWHM*) of the *C*(*r*, *t*) profile. The normalized amount of product *C*, ${\overline{n}}_{C}$, is defined as:

3 $${\overline{n}}_{C}=\frac{{n}_{C}}{\pi h{r}_{max}^{2}{C}_{max}},$$

where *r*_*max*_ = 40 mm is the radius of the HS cells and *C*_*max*_ the maximum value of *C*(*x*, *y*, *t*) observed in the reactor throughout the full experiment duration.

### Numerical Simulations

The model consists of a 2-dimensional axisymmetric system described by the following set of partial differential equations:

4 $${\partial }_{t}A+\left({v}_{r}-\frac{{D}_{A}}{r}\right){\partial }_{r}A={D}_{A}\left({\partial }_{r}^{2}A+{\partial }_{z}^{2}A\right)-kAB$$

5 $${\partial }_{t}B+\left({v}_{r}-\frac{{D}_{B}}{r}\right){\partial }_{r}B={D}_{B}\left({\partial }_{r}^{2}B+{\partial }_{z}^{2}B\right)-kAB$$

6 $${\partial }_{t}C+\left({v}_{r}-\frac{{D}_{C}}{r}\right){\partial }_{r}C={D}_{C}\left({\partial }_{r}^{2}C+{\partial }_{z}^{2}C\right)+kAB$$

Here, *r*, *t*, and *z* represent the dimensional radial coordinate, time, and height, respectively. The functions *A* = *A*(*r*, *z*, *t*), *B* = *B*(*r*, *z*, *t*), and *C* = (*r*, *z*, *t*) denote the dimensional concentrations of the chemical species, and *D*_*A*_, *D*_*B*_, and *D*_*C*_ are their diffusion coefficients. We assume the same mobility for all the species i.e., the diffusivities are set equal to *D*_*A*_ = *D*_*B*_ = *D*_*C*_ = 4 × 10^−10^ m^2^ s^−1^ ^22^. The kinetic constant, *k*, is set to 200 L s^−1^ mol^−1^ ^22^ (leading thus to a Damköhler number, *Da*, with a higher order of magnitude, *Da* > > 1).

Assuming flow incompressibility and radial symmetry, the dimensional advective velocity field, **v**, is a function of the radial component only: **v** = *v*_*r*_(*r*, *z*)**e**_*r*_, where **e**_*r*_ denotes the unit radial vector. Inside the gap of the cell, *v*_*r*_ follows a Poiseuille profile defined along the radial coordinate as^16^:

7 $${v}_{r}(r,z)={v}_{m}(r)\left(1-\frac{4{z}^{2}}{{h}^{2}}\right)$$

and

8 $$\quad {v}_{m}(r)=\frac{3Q}{4\pi hr},$$

where *v*_*m*_(*r*) is the maximum velocity of the flow located at *z* = 0. The simulations are performed within the numerical domain depicted in Fig. 5a, while panel b illustrates the three studied cases as a function of the separation gap height, *h*, leading to different Poiseuille profiles depending on this parameter.

At the horizontal boundaries *z* = − *h*/2 and *z* = *h*/2, a no-slip condition is set for the velocity field (*v*_*r*_ = 0), and a no-flux condition for the chemical species ( − **n** ⋅ ( − ∇ *c*_*i*_) = 0). The boundary at *r* = *r*_0_ = 0.5 mm is designated as inlet for the flow (*v*_*r*_ = *v*_0_) and inflow for the chemical species (*c*_*i*_ = *c*_0,*i*_, *i* = *A*, *C*). The inflow concentrations for the species *A* and *C* are defined by Equation (9), (10) and set to zero for the species *B*. The boundary at *r* = 40 mm is designated as a pressure condition for the flow (*p* = 0) and outflow for the three chemical species (**n** ⋅ ( − ∇ *c*_*i*_) = 0).

To be consistent with the experimental situation, the model accounts for the effect caused by a small premixed concentration of product *C* in the solenoid valve employed in the Hele-Shaw setup (cf. section Sounding Rocket Experiment). For this purpose, two parametric piecewise functions are defined at the inlet boundary:

9 $${C}_{p}=f({r}_{0},z,t,{C}_{p,max},{t}_{C},t{r}_{z}),$$

10 $${A}_{p}=f({r}_{0},z,t,{A}_{p,min},{t}_{C},t{r}_{z})$$

where the function *f* is expressed in terms of the following parameters: *C*_*p*_ and *A*_*p*_ stand for the concentrations of the species *C* and *A* affected by premixing. The function for *B* is implicitly included by conservation, as this species is limiting in the valve reservoir. The parameter *C*_*p*,*max*_ denotes the maximum premixed concentration of the species *C*, and *A*_*p*,*min*_ = *A*_0_ − *C*_*p*,*max*_ represents the concentration of species *A* in the premixed volume, that does not react with *B*. The time span during which *C* is entering with a concentration *C*_*p*,*max*_ is denoted by *t*_*C*_, and *tr*_*z*_ controls the softness of the transition zone until the concentration of the species *C* at the inlet switches from *C*_*p*,*max*_ to zero in the case of *C*_*p*_ (or from *A*_*p*,*min*_ to *A*_0_ in the case of *A*_*p*_). The model is closed with the following initial conditions: *A*(*r* > *r*_0_, *z*, 0) = *C*(*r* > *r*_0_, *z*, 0) = 0 and *B*(*r* > *r*_0_, *z*, 0) = *B*_0_ = 0.03 mol L^−1^.

**Fig. 9:**
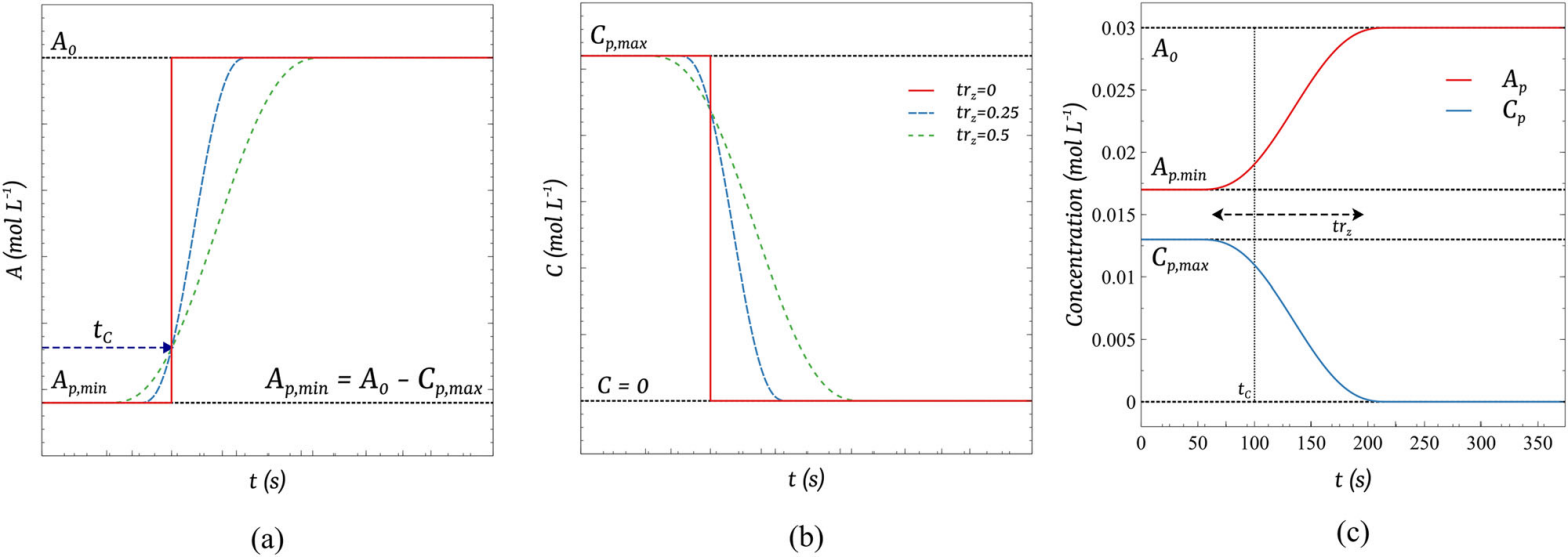
Graphical interpretation of the functions *A*_*p*_ and *C*_*p*_. **a** Plot of generic function *A*_*p*_ and **b** *C*_*p*_ for varied values of transition zone and fixed injection time. **c** Plot of both functions when *t*_*C*_ = 100 s, *tr*_*z*_= 0.5, and the other parameters are set as their experimental counterpart: *A*_0_ = 0.03 mol L^−1^, *C*_*p*,*max*_ = 0.013 mol L^−1^, and *A*_*p*,*min*_ = 0.017 mol L^−1^.

Figure 9 provides a graphical interpretation of the premixing functions defined by Eqs (9), (10). Figure 9a, b show the effect of changing *tr*_*z*_, at fixed *t*_*C*_, on the generic functions *A*_*p*_ and *C*_*p*_, respectively. Figure 9c compares both functions when *t*_*C*_ = 100 *s* and *tr*_*z*_ = 0.5 in a simulated experimental condition.

The premixing factors used for all simulations are included in Table 3.

**Table 3:** Premixing factors used for the three different simulations

Experiment	*h* (mm)	*C*_*p*,*max*_ (mol L^−1^)	*t*_*C*_ (s)	*tr*_*z*_
1	0.2	0.009	5	0.14
2	0.6	0.008	5	0.28
3	1.0	0.013	17	0.25

The PDE systems described by Eqs. (4)-(6) are numerically solved in Comsol Multiphysics 6.0 (COMSOL Inc. Stockholm, Sweden), using the Reacting Flow for Diluted Species (rfd) module. To ensure numerical stability and computational performance, the calculations are done in two simulation steps. In the first one, the solver calculates the stationary Stokes flow defined by the analytical velocity field in Eq. (7). In the second step, it solves the RDA equations of the chemical species advected by the flow calculated in step 1. A sufficiently fine mesh is employed in the numerical domain to ensure grid independence, and the time step is automatically controlled by the software (grid and time step independence studies are provided in the Supplementary Information). Additionally, 1D simulations (not accounting for the sheared Poiseuille profile)^13^ have been conducted for comparison. More details about the 1D simulations are also included in the Supplementary Information.

### Reporting summary

Further information on research design is available in the Nature Research Reporting Summary linked to this article.

### Supplementary information

Supplemental Information

Reporting Summary

## Data Availability

All data used for this publication are uploaded in a data repository (10.14278/rodare.2545) and accessible upon request.

## Code availability

All code used for this publication is available upon reasonable request.
