# Supplementary material for: Unraveling dispersion and buoyancy dynamics around radial A + B → C reaction fronts: microgravity experiments and numerical simulations
Source: NPJ Microgravity. 2024 May 9;10:53. doi: 10.1038/s41526-024-00390-8 (PMC11082159; doi:10.1038/s41526-024-00390-8)
Supplement: Supplementary file 1 — Supplemental Information [file 41526_2024_390_MOESM1_ESM.pdf]

Supplementary Information for: Unraveling dispersion and buoyancy dynamics around radial $A + B \to C$ reaction fronts: microgravity experiments and numerical simulations.

Yorgos Stergiou,[1,2,*] Darío M. Escala,[3,*] Paszkál Papp,[4] Dezső Horváth,[5] Marcus J.B. Hauser,[6] Fabian Brau,[3] Anne De Wit,[3] Ágota Tóth,[4] Kerstin Eckert,[1,2] and Karin Schwarzenberger[1,2]

[1]*Institute of Fluid Dynamics, Helmholtz-Zentrum Dresden-Rossendorf, Bautzner Landstr. 400, 01328 Dresden, Germany*

[2]*Institute of Process Engineering and Environmental Technology, Technische Universität Dresden, 01062 Dresden, Germany*

[3]*Nonlinear Physical Chemistry Unit, Service de Chimie Physique et Biologie Théorique, Faculté des Sciences, Université Libre de Bruxelles (ULB), CP 231, 1050 Brussels, Belgium*

[4]*Department of Physical Chemistry and Materials Science, University of Szeged, Rerrich Béla tér 1., Szeged, Hungary*

[5]*Department of Applied and Environmental Chemistry, University of Szeged, Rerrich Béla tér 1., Szeged, Hungary*

[6]*Faculty of Natural Science, Otto-von-Guericke-Universität Magdeburg, Universitätsplatz 2, 39106 Magdeburg, Germany*

I. SUPPLEMENTARY FIGURES

In Supplementary Figures 1-3, an overview through time of the experiments (1 to 3, respectively) onboard TEXUS 57 is presented.

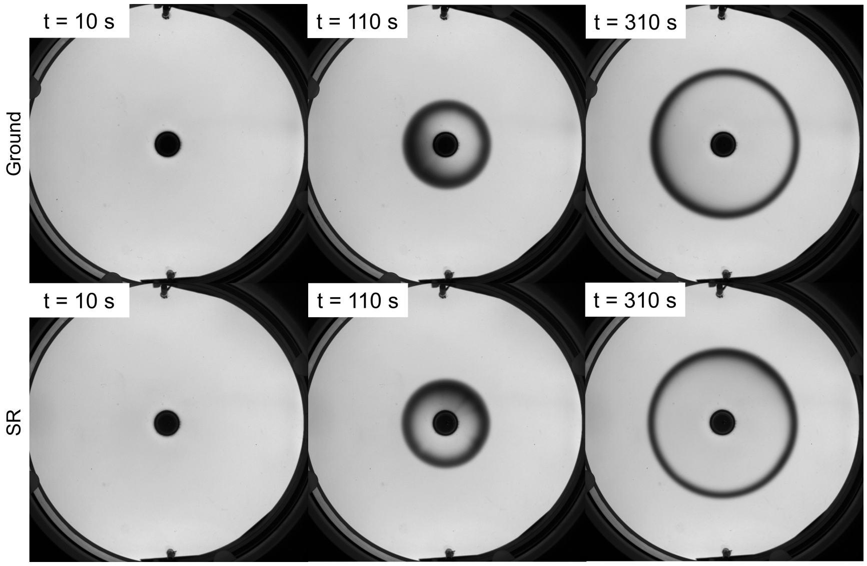

FIG. 1: Temporal evolution of the reaction front in experiments with $h = 0.2$ mm on ground (top) and onboard the SR (bottom).

* Authors contributed equally to this work.

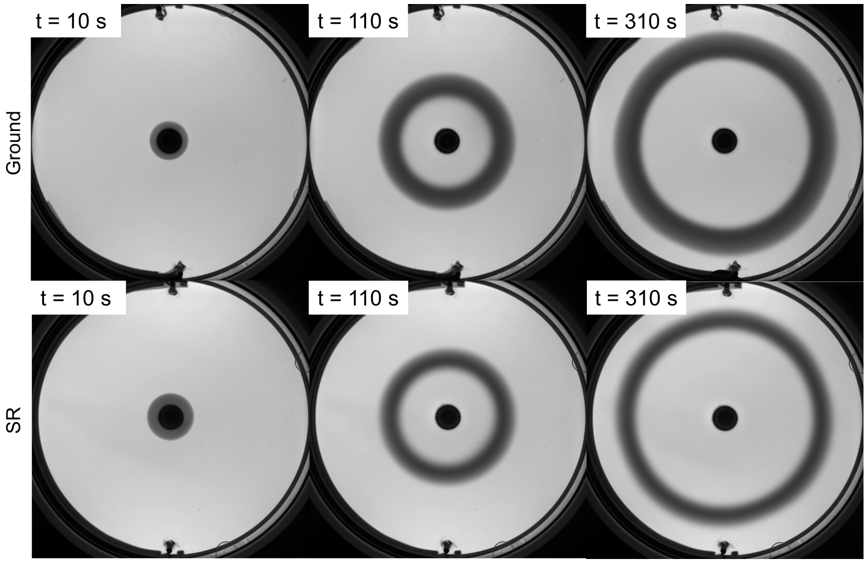

FIG. 2: Temporal evolution of the reaction front in experiments with $h = 0.6$ mm on ground (top) and onboard the SR (bottom).

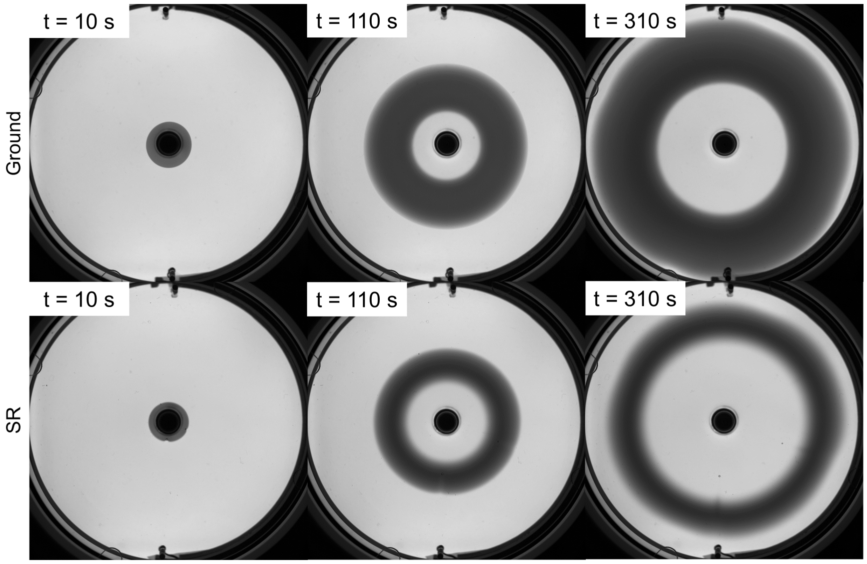

FIG. 3: Temporal evolution of the reaction front in experiments with $h = 1.0$ mm on ground (top) and onboard the SR (bottom).

II. SUPPLEMENTARY METHODS

In order to correlate the normalized grayscale intensity obtained by the camera recordings, I, to the local concentration of product C ($FeSCN^{2+}$), the three different Hele-Shaw reactors were filled with solutions of varied known product concentrations (in 10 steps from 0 to 0.015 mol L^{-1}). The calibration parameters were extracted by fitting the data to a curve of the form:

$$I = I_\infty(1 - e^{-\kappa h C(x,y,t)}), \tag{1}$$

where h is the gap height of the HS reactor and κ, I_∞ are constants. The values that resulted from the concentration calibration of the three different HS cell sizes are presented in Supplementary Table 1. Note that for the HS cell with $h = 0.2$ mm, a Schneider BP HT blue bandpass filter (Jos. Schneider Optische Werke GmbH, Bad Kreuznach, Germany) was used in combination with the camera lens to enhance intensity difference for the thinnest fluid gap.

TABLE 1: Calibration parameters. The discrepancy in the fitting parameters for the 0.2 mm gap height is due to the use of a blue filter in these experiments.

h (mm)	I_∞	κ (L mol^{-1} mm^{-1})
0.2	1.781±0.25	288.3±56.8
0.6	1.183±0.06	218.4±21.2
1.0	1.068±0.03	192.2±13.1

Solving Supplementary Equation 1 and replacing C with the local, time-dependent product concentration, $C(x, y, t)$, yields:

$$C(x, y, t) = (\kappa h)^{-1} \ln \frac{I_\infty}{I_\infty - I(x, y, t)} \tag{2}$$

where $I(x, y, t)$ is the local normalized pixel intensity (i.e grey value) for time t. In Supplementary Figure 4, the calibration curves for all three cells (3 gap heights) are plotted along with the data used for the calibration and the 95% confidence intervals.

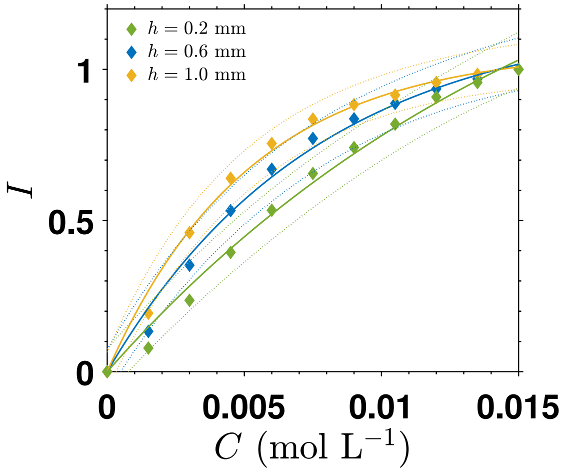

FIG. 4: Calibration curves for the three different HS cells used with $h = 0.2$, 0.6 and 1.0 mm. The data points used for the calibration are plotted along with the fitted curves. The respective dotted lines signify the 95% confidence intervals.

In order to ensure accuracy in the results, a grid independence study was done for each case. Supplementary Figure 5(a-c) compares W_c at $t = t_f$ (final time) as a function of the grid size (number of domain elements N) for each case of study. This observable was chosen as it is particularly sensitive to the premixing functions. Nevertheless, similar results are obtained by testing other observables. The selected grid size value (marked by an arrow in Supplementary Figure 5(a-c)) corresponds to the coarsest grid for which the variation in the observable still is minimal after increasing the number of domain elements. In all cases, the simulations were performed over a mapped grid with five boundary layers to account for the non-slip condition at $z = \pm h/2$. Supplementary Figure 5(d) depicts the entire grid with a closer view of

the region indicated by the red square at the origin of the numerical domain.

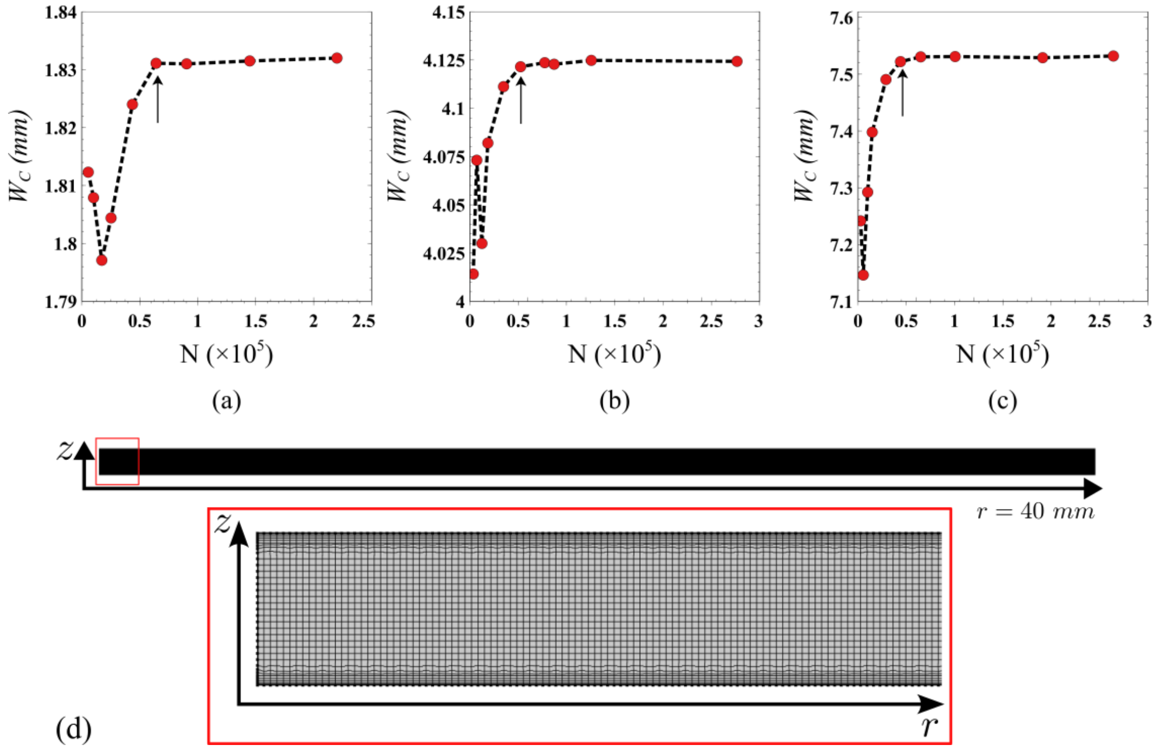

FIG. 5: Grid independence study for the three different h values: 0.2 (a), 0.6 (b), and 1.0 mm (c). N stands for the number of domain elements, and arrows indicate the values selected for each case, which are: 64056 (a), 52462 (b), and 44031 elements (c), respectively. Panel (d) shows a general and a closer view of the numerical grid used in the simulations.

After defining the grid size, the accuracy of the solution was studied by constraining the maximum time step value taken by Comsol's solver. The results are presented in Supplementary Figure 6. In this case, the study was performed on $\bar{n}_C$, but similar results are obtained analyzing other observables. As can be observed, there is no appreciable difference between using a fixed or an automatic time-step setting. For this reason, the automatic time step method is selected over the fixed

one as it significantly reduces the computational time.

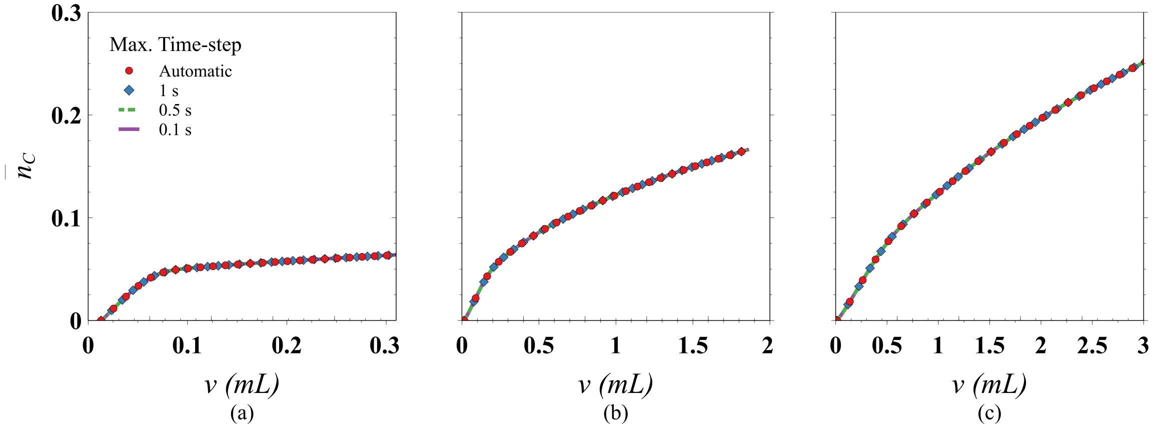

FIG. 6: Time step independence tested for $\bar{n}_C$ at the three different h values: 0.2 (a), 0.6 (b), and 1.0 mm (c). The study compares the results obtained using an automatic time step method with other cases where the maximum time step was constrained, showing no appreciable difference between the methods used.

To verify the effect of the Poiseuille profile in the dynamics of the system, we also simulated the corresponding 1D plug flow model. The equations are given by the following set of PDE:

$$\begin{aligned}
\partial_t A + \left(v_r - \frac{D_A}{r}\right)\partial_r A &= D_A \partial_r^2 A - kAB \\
\partial_t B + \left(v_r - \frac{D_B}{r}\right)\partial_r B &= D_B \partial_r^2 B - kAB \\
\partial_t C + \left(v_r - \frac{D_C}{r}\right)\partial_r C &= D_C \partial_r^2 C + kAB
\end{aligned} \tag{3}$$

where $A = A(r,t)$, $B = B(r,t)$, $C = (r,t)$, and $v_r(r) = \frac{Q}{2\pi hr}$. All other parameters keep the same values as in the 2D case.

The computational domain is defined as the interval $r \in [0.5, 40]$ mm with the boundary conditions at $r = 0.5$ mm and $r = 40$ mm defined identically as for the

2D model. The initial conditions are set as: $A(r > r_0, 0) = C(r > r_0, 0) = 0$ and $B(r > r_0, 0) = B_0 = 0.03$ mol L^{-1}.
The results, with and without premixing, are compared with the corresponding 2D model in the main manuscript.

Two different geometries were used to investigate the effect of imperfect injection: one has a thin tube part below the orifice of the Hele-Shaw (HS) cell (Supplementary Figure 7b), with a length corresponding to the channel between the inlet valve plug and the HS orifice, the other has, in addition, a small gap (1 mm length) below the thin tube with a diameter corresponding to the inlet valve plug (Supplementary Figure 7c).

The effect of product trapped in the tube or the gap, that can cause an elongated injection in the HS cell, is modelled by solving the Navier-Stokes equation with Boussinesq approximation for the reactive mixture on a wedge geometry, with and without gravity, by using the PISO algorithm of the OpenFOAM package with $\Delta t = 0.5$ ms time steps. The concentration dependence of density is based on experimentally determined parameters, the boundary conditions for the concentration field involves setting axisymmetry at the wedge sides and zero gradient at the upper and lower plates. The mesh is divided into $\Delta x = 0.1$ mm and $\Delta z = 0.025$ mm triangular prisms. The calculations are carried out for all three gap height values with both inlet geometries under ground and sounding rocket (microgravity) conditions.
The results of the two cases of premixing geometries are shown in Supplementary Figure 8. It can be seen that the presence of the thin tube alone is not sufficient to reproduce the experimental observation and solution containing product C trapped in a small gap between the inlet valve plug and the inlet tube part can result in the elongated concentration profiles observed experimentally. The effect is most dominant in the HS cell with the smallest gap height ($h = 0.2$ mm). The experimental results lie between these two geometry cases. Additionally, the simulations confirm

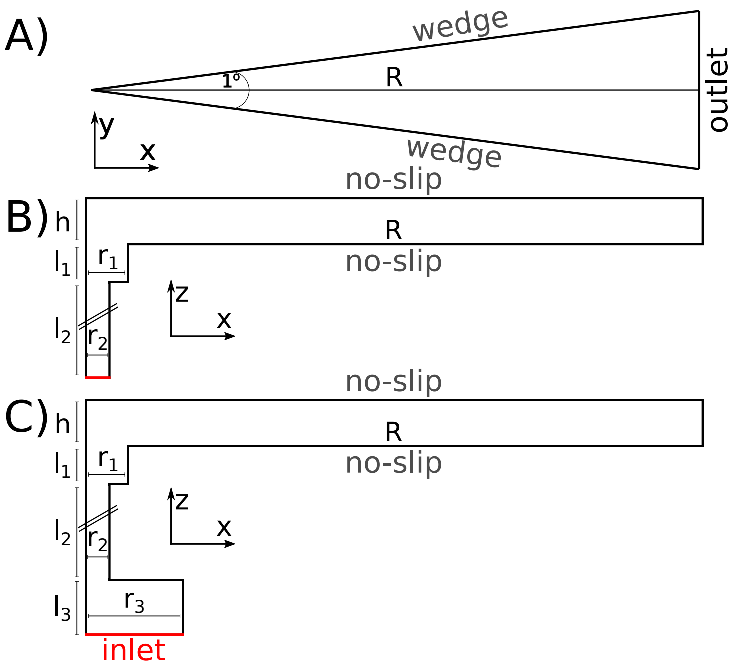

FIG. 7: Reactor geometry with boundary conditions for the velocity field: top view (A) and side view without (B) and with additional gap part (C). The sketches are not drawn to scale for visual clarity. $R = 40$ mm, $r_1 = 0.5$ mm, $r_2 = 0.4$ mm, $r_3 = 3.2$ mm, $l_1 = 4.0$ mm, $l_2 = 42.57$ mm, $l_3 = 1.0$ mm and $h = 1.0, 0.6, 0.2$ mm.

the experimental observation that gravity has only minor effect at $h = 0.2$ mm.

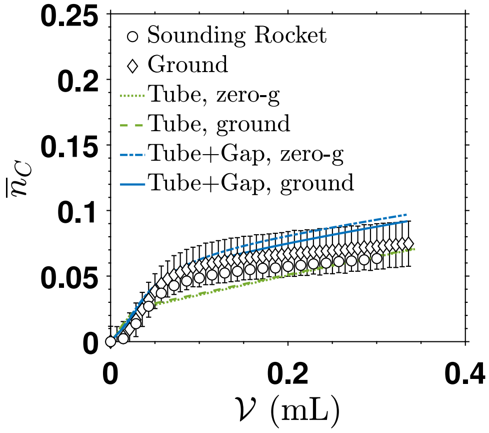

(a)

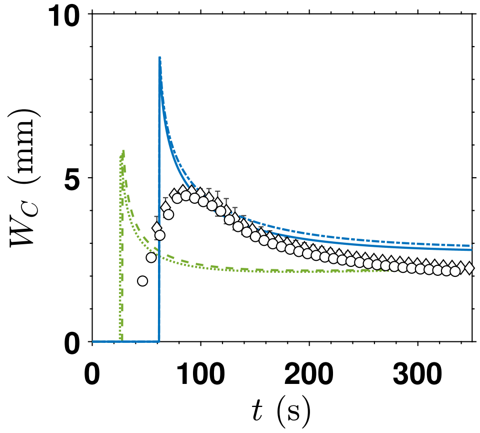

(b)

FIG. 8: Comparison of $\overline{n}_C$ progression with injected volume, $\mathcal{V}$ (a) and W_C progression with time (b) between sounding rocket and ground experiments together with the corresponding numerical simulations for the two different geometries (Tube, Tube+Gap).
